# Elements of Surgical Pathology

**Published:** 1884-01

**Authors:** 


**Elements of Surgical Pathology**. By AUGUSTUS J. PEPPER, M. S., M. B., London, F. R. C. S., England, Fellow of University College, London, Surgeon to St. Mary's Hospital, and Teacher of Practical and Operative Surgery at the Medical School. Illustrated with eighty-one engravings. Henry C. Lea's Son & Co., Philadelphia.

We here also quote from the preface. The author says: "My aim has been to supply what has long been felt as a desideratum, a small work on surgical pathology suited to the requirements of students preparing for their final examinations. The following pages embody some of the conclusions arrived at after many years of study and teaching. An attempt has been made to render in a practical form the knowledge conveyed, and to give an explanation of the causes and methods of pathological processes." We have the same to say of this as of the preceding. It belongs to the same set and will be found a useful little book. It contains 500 pages, and many of the plates are original.

